# Ex-vivo Imaging of Glial Energy Metabolism in the Neonatal Mouse Brain during Convulsive Seizures with Intranasal Radiotracer Administration

**DOI:** 10.1007/s11307-025-02000-9

**Published:** 2025-03-25

**Authors:** Rie Hosoi, Kenya Tada, Takahiro Hayakawa, Yuka Haga

**Affiliations:** Division of Health Sciences, Graduate School of Medicine, University of Osaka, 1-7 Yamadaoka, Suita, Osaka 565-0871 Japan

**Keywords:** Intranasal administration, Neonatal mouse brain, Glia, Pentylenetetrazole, Seizure

## Abstract

**Purpose:**

In this study, we examined changes in glial energy metabolism in neonatal mouse brain images obtained under pathological conditions following intranasal administration of the radiotracer [2-^14^C]acetate.

**Procedures:**

[2-^14^C]acetate was administered via the mouse nasal cavity, after which autoradiograms of the brain of 7-day-old mice were obtained. Radio thin-layer chromatography was applied for metabolite analysis of brain radioactivity. We also compared brain uptake of [2-^14^C]acetate when administrated intranasally and intravenously in 3-week-old mice. To confirm selective uptake by glial cells, [2-^14^C]acetate was injected into the nasal cavity of mice injected with a glial toxin in the brain. Pentylenetetrazole (PTZ) was applied to induce seizures.

**Results:**

Intranasally administered [2-^14^C]acetate was rapidly incorporated into the brains of 7-day-old mice, reaching its highest uptake level 20 min after administration. After 20 min of intranasal [2-^14^C]acetate administration, glutamate and glutamine accounted for 32 ± 2.5% and 30 ± 3.4% of total brain radioactivity, respectively. There was no difference in the radioactivity distribution in the brain between intranasal and intravenous administration, except in the ventral olfactory bulb in 3-week-old mice. Microinjection of the glial-specific toxin fluorocitrate reduced the accumulation of radioactivity in the brain by 60% following intranasal administration in 3-week-old mice. The uptake of [2-^14^C]acetate in the brains of 7-day-old mice significantly decreased 30 min after systemic PTZ administration, suggesting a decrease in energy metabolism in glial cells during seizures.

**Conclusions:**

Quantitative images of biological functions in the neonatal mouse brain can be obtained by intranasal administration. This technique allowed the observation of a decrease in acetate uptake associated with convulsive seizures. The results of this study could be applied to the imaging of biological brain functions and research on neurological disorders using labeled probes in neonatal mice.

## Introduction

Intranasal administration has attracted interest in both clinical and basic animal research as an alternative non-invasive method of drug delivery to the brain that can bypass the blood–brain barrier. This administration route further contributes to the reduction of adverse effects and increasing the efficiency of drug action, by avoiding the first-pass effect. Preclinical studies in mouse models of Alzheimer's disease have shown a higher brain delivery, therapeutic efficacy, and improved safety compared to oral and subcutaneous administration [1, 2]. Smith et al. demonstrated that F-18-labeled insulin is rapidly taken up in the main brain regions involved in processing emotions and memories, using positron emission tomography (PET) imaging in non-human primates [3]. In human studies, naloxone has been shown to rapidly occupy the mu-opioid receptors in the brains of healthy male adult human following intranasal administration based on ^11^C-Carfentanil PET imaging [4].

Given the above results, the intranasal administration route is being explored not only in the context therapeutic drug administration for neurological diseases, but also for labeled probe administration for functional brain imaging [5]. Thorne et al. used autoradiography to show that [^125^I]insulin-like growth factor-I administrated intranasally to rats accumulated in the brain within 30 min and was delivered to the brain along with components associated with olfactory and trigeminal nerves [6]. Singh et al. investigated the feasibility of intranasal administration route in brain PET imaging using [^18^F]fallypride, a potent dopamine D2/D3 receptor antagonist, and blood–brain barrier (BBB) penetrant tracer [7]. They obtained quantitative images of the basal ganglia after intranasal administration and showed that intravenous and intranasal administration resulted in similar uptake into the rat brain. However, as only 0.2% of the administered [^18^F]fallypride reached the basal ganglia, they concluded that the whole brain uptake (excluding the olfactory bulb) of [^18^F]fallypride after intranasal administration is almost completely supplied from the peripheral circulation rather than directly from the nasal cavity. Intranasally administrated fluorescein isothiocyanate-labeled insulin has also been shown to be delivered to the brain by bulk flow in the perivascular lumen within 30 min of nasal administration to rats [8, 9].

This route of administration is also useful for basic studies in animals that cannot be intravenously administered labeled probes. In a mature rodent study, it is technically feasible and routine to inject the labeling probes intravenously into the tail vein. However, intravenous administration in neonatal mice with relatively small tail is difficult. Most animal models of human diseases, including epilepsy, use mature animals. However, the highest incidence of epilepsy occurs during the early age of children [10, 11]. Therefore, the choice of the age of animals should be an important factor, and the use of immature animals would better reflect the actual pathophysiology of epilepsy and provide novel insights. In our present study, 7-day-old mice were selected and examined to obtain *in vivo* functional brain images during pentylenetetrazole (PTZ)-induced seizures. The nasal route was attempted to administer the labeled [2-^14^C]acetate tracer to neonatal mice. Since exogenous acetate is selectively taken up and metabolized by glial cells in the brain [12], the labeled acetate is considered a potential markers of glial cell metabolism. We have previously reported that uptake of labeled acetate is altered in mature animals in a variety of brain disease models, including epilepsy [13, 14].

In the present study, we showed that the intranasal administration of [2-^14^C]acetate to 7-day-old mice could be used to image glial energy metabolism in the brain using autoradiography. We also compared brain uptake of [2-^14^C]acetate when administrated intranasally and intravenously in 3-week-old mice, which are the youngest that can be administered intravenously through tail vein. Then, we further attempted to image metabolic changes in the glial cells of neonatal mice under pathological conditions by imaging glial metabolism in mice with convulsive seizures induced by PTZ administration.

## Materials and Methods

### Ethics Statement

All animal experiments were approved by the Institutional Animal Care and Use Committee, the Division of Health Sciences, Graduate School of Medicine, Osaka University (approval no. 31–03-3).

### Animals

For 7-day-old mice study, pregnant embryonic day 12–13 female ICR mice were obtained from Japan SLC Inc. (Shizuoka, Japan). Experiments were performed when females gave birth to 10–15 pups to avoid variations in growth, because litter size affects the postnatal growth and development. For 3-week-old mice study, 3-week-old male ICR mice were obtained from Japan SLC Inc. (Shizuoka, Japan). All animals were housed under a 12-h light–dark cycle with free access to food and water. Pups were kept warm on a mat at 38 °C during the period when they were separated from their mother.

### Chemicals

[2-^14^C]acetate (specific activity, 1.99 GBq/mmol) was purchased from PerkinElmer Life Science Inc. (Boston, MA, USA). ^14^C-microscale (RPA 511) was obtained from Amersham Biotech UK Ltd. (Buckinghamshire, UK). PTZ was obtained from Sigma-Aldrich (St. Louis, MO, USA). DL-fluorocitric acid barium salt was obtained from Sigma-Aldrich (St. Louis, MO, USA), and prepared as described by Paulsen et al. [15]. All other chemicals used were of the highest commercially available purity.

### Surgery and Microinjection of Fluorocitrate

At 3-week-old, the mice were anesthetized using isoflurane (induction, 5%; maintenance, 2%), and placed in a stereotaxic apparatus. Fluorocitrate was injected into the striatum (0.5 nmol/µL, at a rate of 0.25 µL/min for 4 min) via a 30-gauge cannula using an automated syringe pump. The cannula was left in place for an additional 5 min after injection to reduce the reflux of the injected chemicals along the cannula track. Four hours following fluorocitrate injection, the animals were subjected to [2-^14^C]acetate administration.

### [2-^14^C]Acetate Administration

Seven-day-old animals were maintained in the supine position and intranasally administered 5 µL [2-^14^C]acetate (92.5 kBq/animal) over a period of 1 min by a micropipette. For intranasal administration to 3-week-old mice, animals were placed in an anaesthesia box filled with isoflurane (5%) for 1 min, after which they were removed, held in the supine position, and immediately administrated 5 µL [2-^14^C]acetate (92.5 kBq/animal) intranasally. Following intranasal administration, the animals were kept in the supine position for an additional 1 min, returned to their cages, and allowed to move freely. For intravenous administration, the animals were administrated a bolus injection of [2-^14^C]acetate (92.5 kBq/animal), dissolved in 0.2 mL saline via the tail vein. For oral administration, 7-day-old animals were maintained in the supine position and orally administered 5 µL [2-^14^C]acetate (92.5 kBq/animal) by a micropipette.

### [2-^14^C]Acetate Uptake

In the time course experiment using 7-day-old mice, the animals were sacrificed by decapitation under brief anaesthesia (isoflurane 5%) 5, 20, and 60 min after intranasal administration of [2-^14^C]acetate. In other experiments, 20 min after [2-^14^C]acetate administration, the animals were sacrificed by decapitation under brief anaesthesia (isoflurane 5%). For autoradiography, brains were quickly removed, surrounded with powdered dry ice and frozen. Next, coronal or sagittal slices (20-µm-thick) were prepared using a cryostat at –20 °C, arranged onto glass slides, and placed in contact with an imaging plate (Fuji Film Co., Tokyo, Japan) for several days. The photo-stimulated luminescence (PSL) values in each region of the prepared autoradiograms were subsequently determined using a multipurpose imaging scanner (FLA-7000; Fuji Film Co., Tokyo, Japan). The radioactivity concentrations in the regions of interest (ROIs) were obtained as (PSL-background)/area (mm^2^) [(PSL-BG)/A], calibrated in Bq/g tissue using ^14^C-microscale, and expressed as the distribution absorption ratios (DAR) to correct for differences in animal body weight and injected dose. DAR = radioactivity concentration in tissue (Bq/g) / total injected dose (Bq) × body weight (g). For the dissection study, tissues were quickly removed and weighed, and subsequently solubilised using the tissue solubilizer Soluene-350 (PerkinElmer Co., Ltd. MA, USA). Radioactivity was subsequently measured using a liquid scintillation counter. Radioactivity concentrations are expressed as the DAR values.

### Radio Thin-Layer Chromatography (Radio TLC) Analysis

The animals were sacrificed by decapitation under brief anaesthesia (isoflurane 5%) 20 min after [2-^14^C]acetate intranasal administration. The brains were then quickly dissected, and the cerebral cortex was removed, homogenized with 0.5 N HClO_4_ solution (1:5 w/v), and centrifuged (1000 × g, 10 min). The supernatant was spotted onto thin-layer silica gel plates, and TLC was performed using 99% EtOH:28% NH_3_, 3:1 (v/v) as the eluent. Glutamate and glutamine were spotted on the same TLC plate and used as standard. The TLC plates were exposed to an imaging plate (Fuji Film Co., Tokyo, Japan) for several days. After the autoradiograms were obtained, the TLC plates were sprayed with ninhydrin to determine the Rf value for glutamine and glutamate. Quantitative analysis of the PSL values at each spot was performed using a multipurpose imaging scanner (FLA-7000; Fuji Film Co., Tokyo, Japan), as described above.

### Behavioural Seizure Analysis

Seven-day-old animals were intraperitoneally injected with PTZ (80 mg/kg/25 mL saline) or an equivalent volume of saline. Following this injection, the animals were isolated in plastic cages, and their behaviour was observed for 2 h in 5-min intervals. The presence or absence of any type of behavioural seizure activity was scored as follows: stage 0, no abnormality; stage 1, exploring, and becoming immobilised; stage 2, head nodding, facial and forelimb clonus; stage 3, continuous myoclonic jerk, tail rigidity; stage 4, forelimb and hindlimb clonus, and kangaroo posture; stage 5, tonic–clonic convulsion. These scores align with Racine scale [16] and revised Racine scale applied to mice [17].

### Statistical Analysis

All values are expressed as the mean ± SD (for each group). P-values < 0.05 on repeated measures ANOVA or Student's t-test were considered to indicate statistical significance.

## Results

### Time Course of [2-^14^C]acetate Uptake in Mice Following Intranasal Administration

**Fig. 1:**
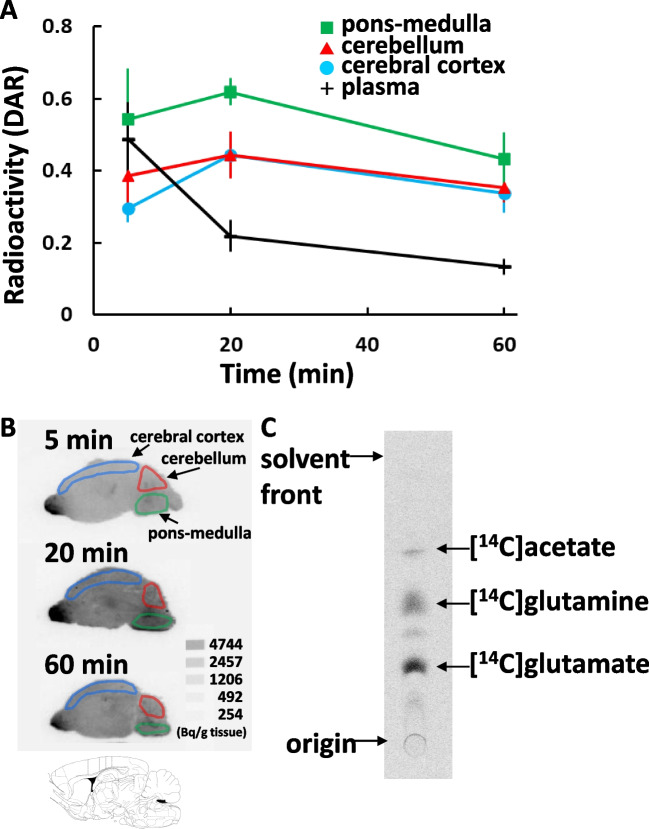
Brain uptake and metabolite analysis following the intranasal administration of [2-^14^C]acetate in 7-day-old mice. **A**: Radioactivity kinetics in 7-day-old mice intranasally administered [2-^14^C]acetate. The radioactivity concentrations are expressed as the DAR (mean ± SD, n = 4 animals per group). **B**: Representative sagittal autoradiograms. The ROIs were identified based on the cerebral cortex, cerebellum, and pons-medulla. **C**: Radio-TLC autoradiogram

Figure 1A shows the kinetics of radioactivity in 7-day-old mice intranasally administered [2-^14^C]acetate. Brain radioactivity was obtained from the autoradiograms (Fig. 1B). which showed that [2-^14^C]acetate was rapidly incorporated, reaching its highest uptake level 20 min after tracer administration. Radioactivity in the pons was significantly higher than that in the cerebral cortex and cerebellum (F[1, 18] = 31.5, p < 0.001, F[1, 18] = 15.8, p < 0.001, respectively). The ventral olfactory bulb showed a high accumulation of radioactivity at all measurement times (Fig. 1B). The radioactivity concentration in the plasma was the highest after 5 min (Fig. 1A).

### Analysis of the Metabolites in Brain Homogenates by TLC

Figure 1C shows the radio TLC autoradiogram of 7-day-old mouse brain homogenates obtained 20 min following the intranasal administration of [2-^14^C]acetate. Glutamate and glutamine accounted for 32 ± 2.5% and 30 ± 3.4% of the total detected radioactivity, respectively (n = 4).

### [2-^14^C]Acetate Uptake in the Brain Following Orally Administration

Brain uptake of [2-^14^C]acetate was 0.414 ± 0.053 and 0.289 ± 0.067 following intranasal and oral administration, respectively (DAR, n = 4, p < 0.05).

### [2-^14^C]Acetate Uptake in the Brain of 3-Week-Old Mice

**Fig. 2:**
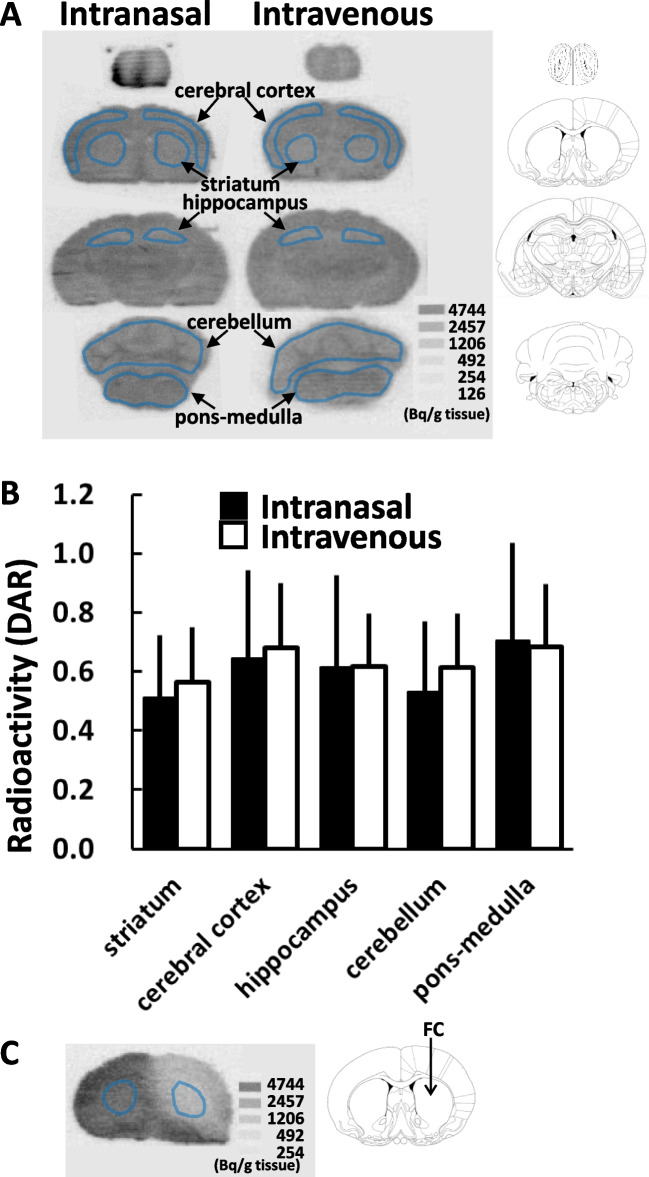
Brain uptake of intranasally and intravenously administered [2-^14^C]acetate in 3-week-old mice. **A**: Representative coronal autoradiograms obtained 20 min following the intranasal or intravenous administration of [2-^14^C]acetate. The ROIs were identified based on the cerebral cortex, striatum, hippocampus, cerebellum, and pons-medulla. **B**: Results of autoradiogram analysis. The radioactivity concentrations are expressed as the DAR (mean ± SD, n = 4 animals per group). **C**: Effect of fluorocitrate (FC) microinjection into the striatum on intranasal administered [2-^14^C]acetate brain uptake

Figure 2 shows the autoradiograms and quantitative results 20 min following intranasal and intravenous administration of [2-^14^C]acetate in 3-week-old mice. There was no difference in radioactivity distribution in the brain between the two administration routes, except in the ventral olfactory bulb. Fluorocitrate microinjection reduced the accumulation of intranasally-administered radiotracer activity to 40.6 ± 4.76% of the contralateral side (n = 4, p < 0.01, Fig. 2C).

### Effects of PTZ on Behaviour in 7-day-old Mice

**Fig. 3:**
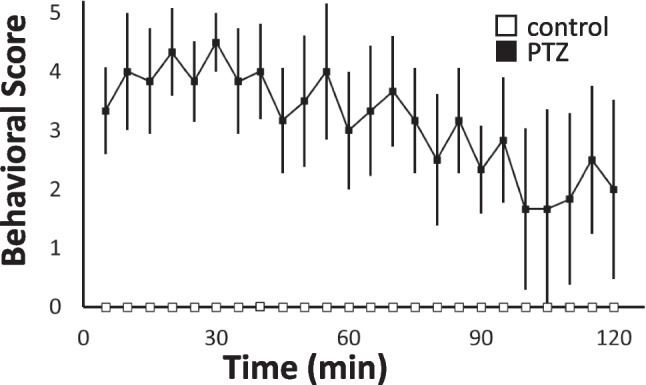
Average behavioural scores over 2 h from 7-day-old mice following treatment with 80 mg/kg PTZ or saline (mean ± SD, n = 6 animals per group)

Figure 3 shows the effects of PTZ on the behaviour of 7-day-old mice. Within 10 min of PTZ administration, mice developed seizures with scores of 4–5. In addition, forelimb and hindlimb clonus, tonic–clonic convulsion with loss of posture continued intermittently 20–60 min after administration. Subsequently, abnormal involuntary movements were observed for more than 1 h. No abnormal behaviours were observed in the saline-treated mice.

### Effects of PTZ on [2-^14^C]Acetate Uptake in Brain

**Fig. 4:**
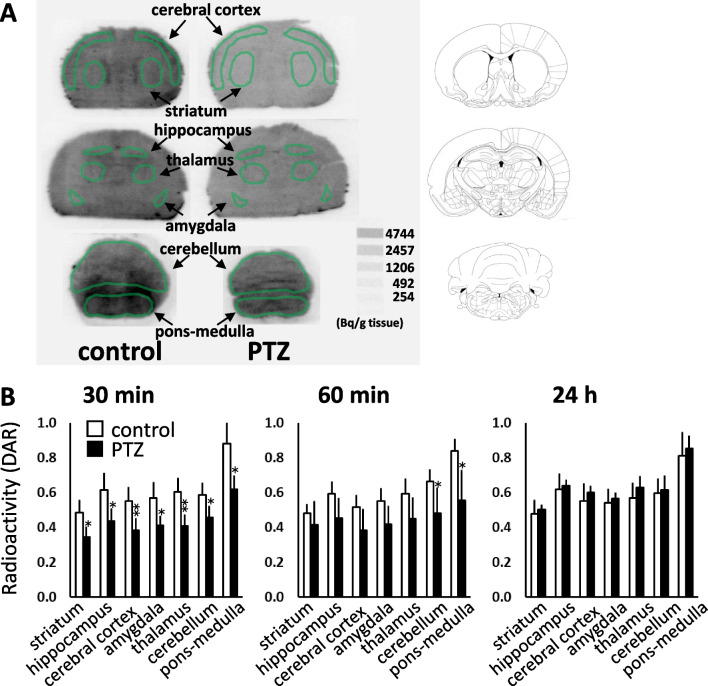
Effect of PTZ on the brain uptake of intranasally administered [2-^14^C]acetate in 7-day-old mice. **A**: Representative coronal autoradiograms 30 min following PTZ administration. The ROIs were identified based on the cerebral cortex, striatum, hippocampus, thalamus, amygdala, cerebellum, and pons-medulla. **B**: Results of autoradiogram analysis. The radioactivity concentrations are expressed as the DAR (mean ± SD, n = 5 animals per group, *P < 0.05, **P < 0.01 vs control)

Figure 4A shows representative images of [2-^14^C]acetate uptake in the brain 30 min after PTZ administration. Figure 4B shows the quantitative results of the images obtained at 30 and 60 min, and 24 h after PTZ administration. [2-^14^C]acetate uptake in the whole brain was reduced to 70% of that in the control group 30 min after PTZ administration, with uptake gradually recovering to control levels after 24 h.

### Effects of PTZ on [2-^14^C]Acetate Uptake in Peripheral Tissue

**Fig. 5:**
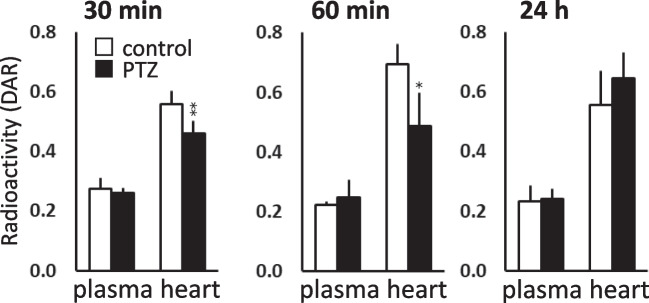
Effect of PTZ on the peripheral tissue uptake of intranasally administered [2-^14^C]acetate in 7-day-old mice. The radioactivity concentrations are expressed as the DAR (mean ± SD, n = 5 animals per group, *P < 0.05, **P < 0.01 vs control)

The [2-^14^C]acetate uptake in the heart was reduced to 80% of that in the control group at 30 min, and 70% at 60 min after PTZ administration (Fig. 5). This decrease was reversed at 24 h. No changes in plasma radioactivity levels were observed following PTZ treatment.

### Effects of PTZ on Body Weight

After seizures had resolved, the pups were returned to their cage and reared by their mother in the same manner as control pups. The body weight at 24 h after PTZ administration was 4.48 ± 0.59 g in the control group (n = 5) and 4.40 ± 0.63 g in the PTZ group (n = 5), while the body weight gains of these groups were 0.51 ± 0.17 g and 0.35 ± 0.14 g, indicating that the PTZ group showed a weight suppression tendency.

## Discussion

Functional brain imaging using labeled probes is widely used in both clinical and basic research and is also applied to animal model studies of human diseases. Most of these animal models use mature animals. However, there are diseases that occur at a high rate during a specific period, such as epilepsy, which has a high incidence during the early age of childhood [10, 11]. Age matching animal models might have the potential to provide new insights into disease pathology. In this study, we investigated to obtain functional brain images of neonatal mice during convulsive seizures. For neonatal mice, intranasal administration of labeled probe was applied instead of intravenous administration. Considering the invasiveness and technical issues in neonatal mice, experiments on the comparisons of intravenous and intranasal administration of labeled probes and the microinjection study were performed on 3-week-old mice, instead of 7-day-old mice.

Intranasally-administered [2-^14^C]acetate showed high brain uptake in 7-day-old mice (Fig. 1A, B). In 7-day-old mice, more than 60% of the radioactivity in the brain was converted to carbon-14 labeled glutamate and glutamine (Fig. 1C). Prior reports have indicated that intravenous or intraperitoneal administration of carbon-14 labeled acetate is converted to labeled glutamate and glutamine within minutes [18–20]. Acetate enters glial cells through monocarboxylate transporter 1, which is highly expressed in glia [21] and is metabolized by acetyl coenzyme A (acetyl-CoA) synthetase to form acetyl-CoA, which enters the tricarboxylic acid (TCA) cycle. Acetyl-CoA is then used in the production of adenosine triphosphate (ATP) and in the synthesis of lipids and neurotransmitters. Glutamate is derived from alpha-ketoglutarate, which is an intermediate in the TCA cycle. Glutamate can be directly converted to glutamine by the glutamine synthetase that is exclusively located in glia. Glutamine is transferred to glutamatergic neurons to serve as precursor for glutamate or for the TCA cycle intermediates. In this study, intranasally administered [2-^14^C]acetate is assumed to have reached into the brain and acted as a metabolic substrate.

In 3-week-old mice, there was no difference in radioactivity distribution in the brain between the two administration routes, except in the ventral olfactory bulb (Fig. 2A, B). We found that the brain uptake of intranasally-administered [2-^14^C]acetate was effectively inhibited by microinjection of the glial-specific toxin fluorocitrate (Fig. 2C). Fluorocitrate is a selective inhibitor of aconitase in the glial TCA cycle. Intrastriatal injection of 1 nmol fluorocitrate transiently causes a severe reduction in glutamine levels 4 h after injection [15]. Under these conditions, [2-^14^C]acetate uptake was reduced by more than 50% in mature rat brains [22]. The decrease in the uptake of intranasally administered [2-^14^C]acetate is suggested to reflect the decrease in the metabolism of the glial TCA cycle.

Based on these results, we successfully obtained brain images reflecting glial cell metabolism from intranasally-administered [2-^14^C]acetate and ex-vivo functional brain images of 7-day-old mice. To the best of our knowledge, this study is the first report to show the ex-vivo functional brain images of 7-day-old mice using intranasal administration.

Intranasally administered labeled probes are rapidly transported into the brain via two distinct extracellular transport pathways associated with the olfactory and trigeminal nervous systems, as well as via bulk flow within the cerebral perivascular spaces [6, 8, 9, 23, 24]. The presence of nucleoside transporters may play an important role in the nasal-to-brain delivery of certain nucleosides such as [^18^F]-fluoro-3'-deoxy-3'-L-fluorothymidine [25]. The systemic pathway is also an indirect route from the nose to the brain [26]. The exact mechanisms of this nose-to-brain delivery are not fully understood, a combination of these pathways is responsible. In rodents, high delivery to the brain was observed 30 min after intranasal probe administration, and our results are consistent with those of previous reports. Because [2-^14^C]acetate is a highly BBB permeable molecule, following intranasal administration, [2-^14^C]acetate may be delivered to the brain directly via the nose and systemic circulation. In this study, we administered the same radioactivity and volume of [2-^14^C]acetate intranasally or orally to 7-day-old mice, and compared brain uptake. Brain uptake of [2-^14^C]acetate after oral administration was 70% of that after intranasal administration. Although intranasally-administered [2-^14^C]acetate could enter the blood through the nasal cavity, subsequently entering the brain from systemic circulation, intranasally administered [2-^14^C]acetate was more efficiently transferred to the brain.

We also showed that PTZ transiently-induced convulsive seizures (Fig. 3) and significantly reduced [2-^14^C]acetate uptake in the brains of 7-day-old mice (Fig. 4). A single dose of PTZ (80 mg/kg, ip) in neonatal mice has been reported to induce repetitive seizures for 2–3 h [27, 28]. Our results suggested that PTZ-induced convulsive seizures caused a transient decrease in glial cell energy metabolism. Changes in cerebral blood flow and other energy substrates, such as glucose metabolism, during PTZ-induced convulsive seizures are important issues for further studies. [2-^14^C]acetate is taken up by the heart as an energy substrate; our investigation showed that PTZ-induced convulsive seizures also reduced the cardiac energy substrate uptake (Fig. 5). Consistent with the tendency of PTZ to suppress weight gain, persistent convulsive seizures may decrease the uptake and metabolism of energy sources and cause alterations in blood flow throughout the body.

## Conclusions

Overall, the present study showed that quantitative images of biological functions in the neonatal mouse brain can be obtained following the intranasal administration of radiotracers. Furthermore, we observed a decrease in acetate uptake associated with convulsive seizures. The results of this study can also be applied to the imaging of biological brain functions and research on neurological disorders using labeled probes in neonatal mice.

## Data Availability

The datasets generated and/or analysed in this study are available from the corresponding author, Rie Hosoi, upon reasonable request.
